# Data and alternative models describing the associations among non-infection pandemic stress, event-related rumination, depression, and anxiety

**DOI:** 10.1016/j.dib.2022.108864

**Published:** 2022-12-27

**Authors:** Mianzhi Hu, Scott D. Squires, Roumen V. Milev, Jordan Poppenk

**Affiliations:** a Department of Psychology, Queen's University, Kingston, Ontario, Canada; b Centre for Neuroscience Studies, Queen's University, Kingston, Ontario, Canada; c Department of Psychiatry, Queen's University, Kingston, Ontario, Canada; d School of Computing, Queen's University, Kingston, Ontario, Canada

**Keywords:** Deliberate rumination, Intrusive rumination, COVID-19 pandemic, Stress, Depression, Anxiety

## Abstract

Here we present cross-sectional data collected from 1507 participants through the Qualtrics online survey platform. Participants were recruited from Reddit, Facebook, and the Queen's University undergraduate participant pool, and were instructed to complete a pandemic stress survey, the Beck Depression Inventory-II (BDI-II) [1], the Beck Anxiety Inventory (BAI) [2], a modified version of Event-Related Rumination Inventory (ERRI) [3], and a demographics questionnaire. For the 1069 participants who were not exposed to COVID-19 infection, we calculated the sum of each scale/subscale and performed a multiple mediation analysis using MPlus. The results indicated that three models (one primary model and two alternative models) had comparable statistical power to explain the variance as we tested different configurations of predictor, mediator, and outcome variables. Given the cross-sectional nature of the present study, we could not conclude which model was most valid. Therefore, we share our original data and tested models here for others to use. They are useful for researchers who wish to replicate our results, conduct new analyses with these data, or design future studies.

**Specifications Table**

**Table:** 

Subject	Clinical Psychology
Specific subject area	Pandemic stress, internalizing psychopathology, and event-related rumination
Type of data	Table Figure
How the data were acquired	Data were collected with an online survey comprised of the Beck Depression Inventory-II (BDI-II) [1], the Beck Anxiety Inventory (BAI) [2], a modified version of the Event-Related Rumination Inventory (ERRI) [3], and a questionnaire of pandemic stress. We used the Qualtrics online survey platform to collect and filter these data.
Data format	Filtered Analyzed
Description of data collection	Participants were recruited from social media platforms (e.g., Reddit, Facebook) and Queen's University's undergraduate participant pool. They voluntarily answered the survey on their phone or computer using the Qualtrics online survey platform. In the analyzed dataset, we excluded 438 participants who indicated that they or their family members had a history of COVID-19 infection.
Data source location	Institution: Queen's University City/Town/Region: Kingston Country: Canada
Data accessibility [4]	Repository name: Mendeley Data Data identification number: 10.17632/2f6t9pff4n Direct URL to data: https://data.mendeley.com/datasets/2f6t9pff4n
Related research article	Squires, S., Hu, M., Milev, R.V., Poppenk, J., The impact of non-infection pandemic stress on depression and anxiety severity: Investigating mediation by intrusive and deliberate rumination, J. Affect. Disord. *310 (2022) 291-295.*

## Value of the Data

• Due to the cross-sectional nature of these data, the alternative models are useful for considering other, equally plausible explanations of our data besides the main model we explored in Squires et al. [5].
• Researchers working on the dissociation of intrusive versus deliberate rumination may benefit from these data since our models showed a consistent dissociation between intrusive and deliberate rumination no matter where they are located in the model.
• Our data may also benefit scholars who are interested in the psychological impact of public health crises more broadly. These data may be compared to and integrated with other COVID-19, pandemic-related, or other public health datasets.
• Researchers who would like to conduct longitudinal studies on the mediation effects of event-related rumination may be informed by these data. Data presented here encourage future studies to examine stress, event-related rumination, and internalizing psychopathology in a longitudinal design to directly test the direction of causality.
• Finally, this dataset can inform clinical research that aims to treat depression through techniques that target rumination, such as by redirecting ones’ intrusive, unconstructive ruminative thoughts into more deliberate, constructive forms [6].

## 1 Data Description

The following files are available in the Mendeley Data online repository:

**dataset_filtered_n1507.sav:** An SPSS file that contains dataset collected from 1507 participants who completed all items of the COVID-19 Impact Scale, ERRI, BDI-II, and BAI. Includes participants who experienced COVID-19 infection themselves or among family. Only fully complete data is presented here. Incomplete (not 100% complete) data has been filtered out. Table 1 summarizes the demographic characteristics of this COVID-infected sample.

**Table 1:** Demographic and clinical information of the COVID-infected sample.

Demographic covariates	Percentage (*n* = 1507)
**Age in years**	
18-25	65.2
26-35	23.0
36-45	7.5
46-55	2.3
>55	1.8
**Gender**	
Male	25.3
Female	74.0
Other	0.6
**Race/Ethnicity**	
Caucasian	63.4
Asian	20.7
African	2.2
Hispanic	2.8
Other	10.9
**Marital status**	
Single (never married)	54.5
Dating	23.4
Common law	4.2
Married	13.7
Divorced/Separated	2.5
Other	1.5
**Occupational status**	
Student	55.1
Unemployed	8.0
Part-time	16.5
Full-time	27.5
Other	6.8
**Depression Severity**	
None	44.3
Mild	16.1
Moderate	18.5
Severe	21.0
**Anxiety Severity**	
None	44.3
Mild	25.2
Moderate	17.3
Severe	13.1

**dataset_filtered_n1507_CSV.csv:** The previously described filtered dataset, but in comma-separated format.

**dataset_analyzed_n1069.sav:** An SPSS file containing the dataset used in our multiple mediation analysis, collected from 1069 participants. Includes all participants in filtered dataset except those with experience of COVID-19 infection, whether themselves or among family. Table 2 summarizes the demographic characteristics of this COVID-free sample.

**Table 2:** Demographic and clinical information of the COVID-free sample.

Demographic covariates	Percentage (*n* = 1069)
**Age in years**	
18-25	68.5
26-35	20.8
36-45	7.0
46-55	1.9
>55	1.9
**Gender**	
Male	24.9
Female	74.6
Other	0.6
**Race/Ethnicity**	
Caucasian	63.3
Asian	20.9
African	2.4
Hispanic	1.8
Other	11.6
**Marital status**	
Single (never married)	57.5
Dating	23.9
Common law	4.3
Married	11.0
Divorced/Separated	2.0
Other	1.3
**Occupational status**	
Student	58.9
Unemployed	8.0
Part-time	16.3
Full-time	24.1
Other	7.3
**Depression Severity**	
None	45.7
Mild	16.1
Moderate	17.6
Severe	20.7
**Anxiety Severity**	
None	47.8
Mild	26.6
Moderate	15.1
Severe	10.1

**dataset_analyzed_n1069_MPlus.dat:** An MPlus-compatible dataset that serves as one of the input files for the multiple mediation models. Contains only the following variables: Age, Sex, COVID-19 Impact total, Intrusive Rumination (ERRI) total, Deliberate Rumination (ERRI) total, BDI-II total, and BAI total.

**dataset_analyzed_n1069_MPlus_headers.csv:** The headers for the columns in “dataset_analyzed_n1069_MPlus.dat”. (For reference only).

**ItemCodes.docx:** The descriptions of each item and their associated ID codes for the dataset files.

**Questionnaires.docx:** The instructions and item lists for the COVID-19 Impact Scale, ERRI, BDI-II, and BAI.

**model1.txt:** The MPlus input file that defines our main multiple mediation model, which was investigated in Squires et al. (2022) (Model 1). In this model, the predictor is COVID-19 Impact Scale total score, the outcomes are BDI-II and BAI total scores, and the mediators are Intrusive Rumination and Deliberate Rumination subscale scores; age and sex are included as covariates.

**model2.txt:** The MPlus input file that defines an alternative model to Model 1. In this model (Model 2), the predictor is COVID-19 Impact Scale total score, the outcomes are Intrusive and Deliberate Rumination subscale scores, and the mediators are BDI-II and BAI total scores; age and sex are included as covariates.

**model3.txt:** The MPlus input file that defines and tests another alternative model. In this model (Model 3), the predictors are BDI-II and BAI total scores, the outcome is COVID-19 Impact Scale total, and the mediators are Intrusive Rumination and Deliberate Rumination subscale scores; age and sex are included as covariates.

**results_models2+3.docx:** A summary of the multiple mediation results for Models 2 and 3. The results of Model 1 are presented in Squires et al. [5].

## 2 Experimental Design, Materials and Methods

1507 participants were recruited from Reddit, Facebook, and the Queen's University undergraduate psychology participation pool as part of a larger study about rumination. Data were collected via the Qualtrics survey platform.

The questionnaire started with demographic questions asking participants about their age, gender, ethnicity, native language, marital status, occupational status, medication, etc. Then they were presented with a modified version of the Event-Related Rumination Inventory (ERRI) [3]. The ERRI includes 20 items, 10 for each of its two subscales: deliberate rumination (e.g., “I think about what the event might mean for my future.”) and intrusive rumination (e.g., “Thoughts about the event come to mind and I cannot stop thinking about them.”). Item scores range from 1 (Not at all) to 4 (Often). For the ERRI in this dataset, “…the event…” was replaced by “…the pandemic and its consequences…” to assess rumination about the COVID-19 pandemic specifically. The scores of each subscale were summed up to represent the level of engagement in the corresponding type of rumination.

ERRI was followed by a survey of pandemic-related stress, the COVID-19 Impact Scale, which has four domains – infection of oneself or family, social restrictions, impact on daily life (e.g., employment, education), and shortage of resources (e.g., food, medicine, etc.). Each item's score ranged from 1 (No Impact) to 5 (Severe Impact). Great internal consistency was shown in our sample (Cronbach's α = .82) for this scale. For the analyzed dataset, participants’ data were excluded if the participant indicated that they or a family member had ever been infected by COVID-19. A total of 438 participants reported that they or a family member had a history of COVID-19 infection, and thus data from 1069 participants remained in the analyzed dataset. Two total scores were calculated for this scale by summing up all the relevant items (and are present in the filtered dataset): one that included the two items about personal and family COVID-19 infection, and another that excluded these items. The total with these items removed was what we used in our multiple mediation analyses.

Lastly, the Beck Depression Inventory-II (BDI-II) [1] and the Beck Anxiety Inventory (BAI) [2] were used to measure the severity of depression and anxiety respectively. The BDI-II contains 21 items, with the scores ranging from 0 (the least severe) to 3 (the most severe). It evaluates cognitive, affective, and behavioral changes in the last two weeks (e.g., worthlessness, sadness, changes in appetite). Similarly, BAI also has 21 items ranging from 0 (Not at all) to 3 (Severely). It concerns psychological and physical fluctuations such as numbness, dizziness, heart pounding, sweating, etc. The sum of the scores on each scale represented the severity of depression/anxiety.

None of the items required recoding. Altogether, we calculated five total scores to use as variables in our multiple mediation models: non-infection pandemic stress (PS), deliberate rumination (DR), intrusive rumination (IR), depression severity, and anxiety severity. Age and gender were included as covariates. Our multiple mediation models were tested using MPlus statistical modeling software.

## Ethics Statements

This study was approved by the General Research Ethics Board at Queen's University (GPSYC-765-16). We confirm that informed consent was obtained from participants and that participant data has been fully anonymized. Participants were informed with a letter of information presented immediately after clicking the study link. Participants indicated their consent to participate by advancing to the next page of the online survey (and checking the Captcha box in the process).

## Funding

This work was supported by a 10.13039/501100000038Natural Sciences and Engineering Research Council of Canada (NSERC) Postgraduate Scholarship (Doctoral), awarded to Scott Squires, M.Sc.; and an NSERC Discovery Grant (03637), awarded to Jordan Poppenk, Ph.D.

## CRediT authorship contribution statement

**Mianzhi Hu:** Writing – original draft, Formal analysis, Visualization. **Scott D. Squires:** Conceptualization, Methodology, Validation, Writing – review & editing. **Roumen V. Milev:** Supervision, Methodology. **Jordan Poppenk:** Supervision, Methodology.

## Declaration of Competing Interest

The authors declare that they have no known competing financial interests or personal relationships that could have appeared to influence the work reported in this paper.

## Data Availability

COVID-19 Impact Scale, Event-Related Rumination Inventory, Beck Depression Inventory, and Beck Anxiety Inventory Data from Online Sample (Original data) (Mendeley Data).
